# Factors that influence adult neurogenesis as potential therapy

**DOI:** 10.1186/s40035-018-0109-9

**Published:** 2018-02-21

**Authors:** Belal Shohayeb, Mohamed Diab, Mazen Ahmed, Dominic Chi Hiung Ng

**Affiliations:** 1 School of Biomedical Science, Faculty of Medicine, University of Queensland, St Lucia, QLD 4067 Australia; 2 Faculty of Pharmacy, Pharos University in Alexandria, P.O. Box Sidi Gaber, Alexandria, 21311 Egypt

**Keywords:** Niche, Neurotrophins, Cytokines, Transcription factors, Stem cells, Extrinsic factors, Therapy

## Abstract

Adult neurogenesis involves persistent proliferative neuroprogenitor populations that reside within distinct regions of the brain. This phenomenon was first described over 50 years ago and it is now firmly established that new neurons are continually generated in distinct regions of the adult brain. The potential of enhancing the neurogenic process lies in improved brain cognition and neuronal plasticity particularly in the context of neuronal injury and neurodegenerative disorders. In addition, adult neurogenesis might also play a role in mood and affective disorders. The factors that regulate adult neurogenesis have been broadly studied. However, the underlying molecular mechanisms of regulating neurogenesis are still not fully defined. In this review, we will provide critical analysis of our current understanding of the factors and molecular mechanisms that determine neurogenesis. We will further discuss pre-clinical and clinical studies that have investigated the potential of modulating neurogenesis as therapeutic intervention in neurodegeneration.

## Background

Neurogenesis is an endogenous process that involves coordinated proliferation, differentiation, and migration of neural precursor cells [1]. It determines brain formation during embryonic development and persists in localized regions of the adult brain or neurogenic niches. As a result of aging, brain injury, and genetic mutations, the progressive loss of structure, function, and depletion of neural precursors may contribute to neurodegenerative disorders including Alzheimer’s disease (AD) and Parkinson’s diseases (PD) [2]. For years, the occurrence of neurogenesis in the adult brain and the capacity to generate new neurons has been debated [3, 4], however, a number of studies have provided clear evidence of neurogenesis in the subgranular zone (SGZ) and subventricular zone (SVZ) of the adult brain [1, 5]. At the SVZ, neural stem cells (NSCs) migrate along the rostral migratory stream (RMS) and differentiate into interneurons in the olfactory bulb (OB). NSCs located in the SGZ give rise to granular neurons that integrate into functional circuits in the hippocampus [6]. Studies have revealed important determinants that enhance neurogenesis in the adult brain. These determinants broadly include intrinsic and extrinsic factors. The intrinsic factors include neurotrophic factors [7], transcriptional programs [8], inflammatory cytokines [9], neurotransmitters and hormones [10]. The extrinsic factors include physical activity [10], dietary intake [11] and stem cell transplantation [12]. This review will discuss our current state of understanding of how these factors regulate adult neurogenesis and their potential application towards neurorestorative approaches.

### Intrinsic factors that regulate adult neurogenesis

#### Neurogenic niches impact on neurogenesis

The neurogenic niche represents a specialized microenvironment that has a major role in maintaining and regulating NSC proliferation [6]. Interestingly, several intrinsic factors, such as hormones, trophic factors, glia, and vasculature, contribute to the neurogenic niche. For instance, studies have highlighted the importance of the vasculature in supporting NSCs population, including those in the adult mammalian brain [13]. The first study that reported a role of the vascular niche in regulating neurogenesis revealed a seasonal increase in testosterone levels in male songbirds that were associated with peak neuron replacement in the telencephalon. Increased testosterone release stimulated angiogenesis, which then increased production of brain-derived neurotrophic factor (BDNF). This trophic factor consequently induced the formation of new neurons for the seasonal expansion of the high vocal centers of songbirds [14].

In the brain, blood vessels are in a close proximity to NSCs which are collocated with endothelial cells, crowded at the tips of capillaries in the adult SVZ. Endothelial paracrine signalling and cell-cell contacts facilitate cross-talk with NSCs and are likely involved in integrating neurogenesis and angiogenesis [15]. For example, the contact between endothelial cells and NSCs lining the ventricles is critical for maintaining stemness and the activation of Notch signaling [16]. In-vitro, Notch signaling was upregulated, in a co-culture of neuroepithelial and endothelial cells, which induced the self-renewal of neuroepithelial cells [17]. In addition, the expansion of neuroprogenitor cells upon contact with the co-cultured endothelial cells induced β-catenin signaling [17, 18], which is essential for the formation of cell-cell junctions between neuroprogenitor cells. Disruption of β-catenin leads to instability in neuroprogenitor cell junctions and is associated with a reduction in proliferation and cortical malformation [19, 20]. This indicates that the junctions between neuroprogenitor cells are likely to be essential for maintaining cell proliferation.

In addition, astrocytes in SGZ and SVZ are integral for the adult neurogenic niche, due to their contributions towards promoting proliferation and differentiation of NSCs. Astrocytes further provide a physical support for newborn neurons and facilitate their migration and integration into neuronal circuits [21]. In the neurogenic niche, astrocytes express ciliary neurotrophic factor (CNTF), while the receptor, CNTFRα, is predominantly expressed in neural progenitor cells and hippocampal neurons [22]. CNTF has an essential role in regulating neurogenesis as it maintains NSCs differentiation and proliferation [23, 24]. Taken together, these studies emphasize the significance of the vasculature for the neurogenic microenvironment and the importance of astrocyte and endothelial cells in providing NSCs with trophic factors and physical support. Therefore, a complex interplay of distinct signaling effectors within the neurogenic niche serve to maintain neurogenesis through to adulthood.

#### Neurotrophic growth factors mediate adult neurogenesis

**Fig. 1:**
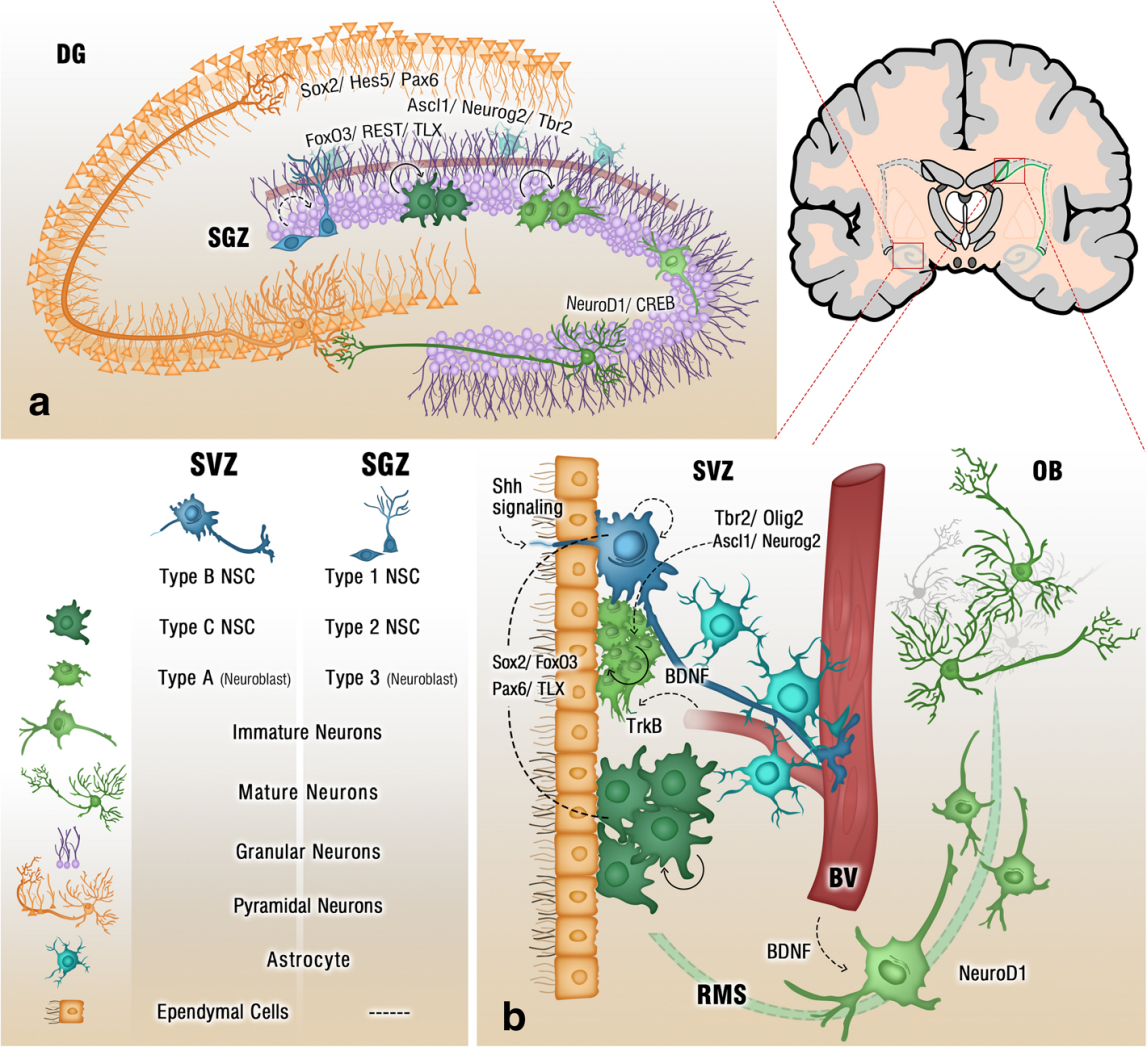
Adult neurogenic niche. Cross section of the adult brain showing regions of SGZ and SVZ, where neurogenesis takes place. The schematic illustrates neurogenesis involving NSCs development into mature neurons and the neurogenic niches of Blood Vessels (BV), astrocytes and cilia, as well as transcription programs in the SGZ (**a**) and SVZ (**b**). Neuronal migration from the SVZ to the OB via the RMS is also shown in (**b**)

**Fig. 2:**
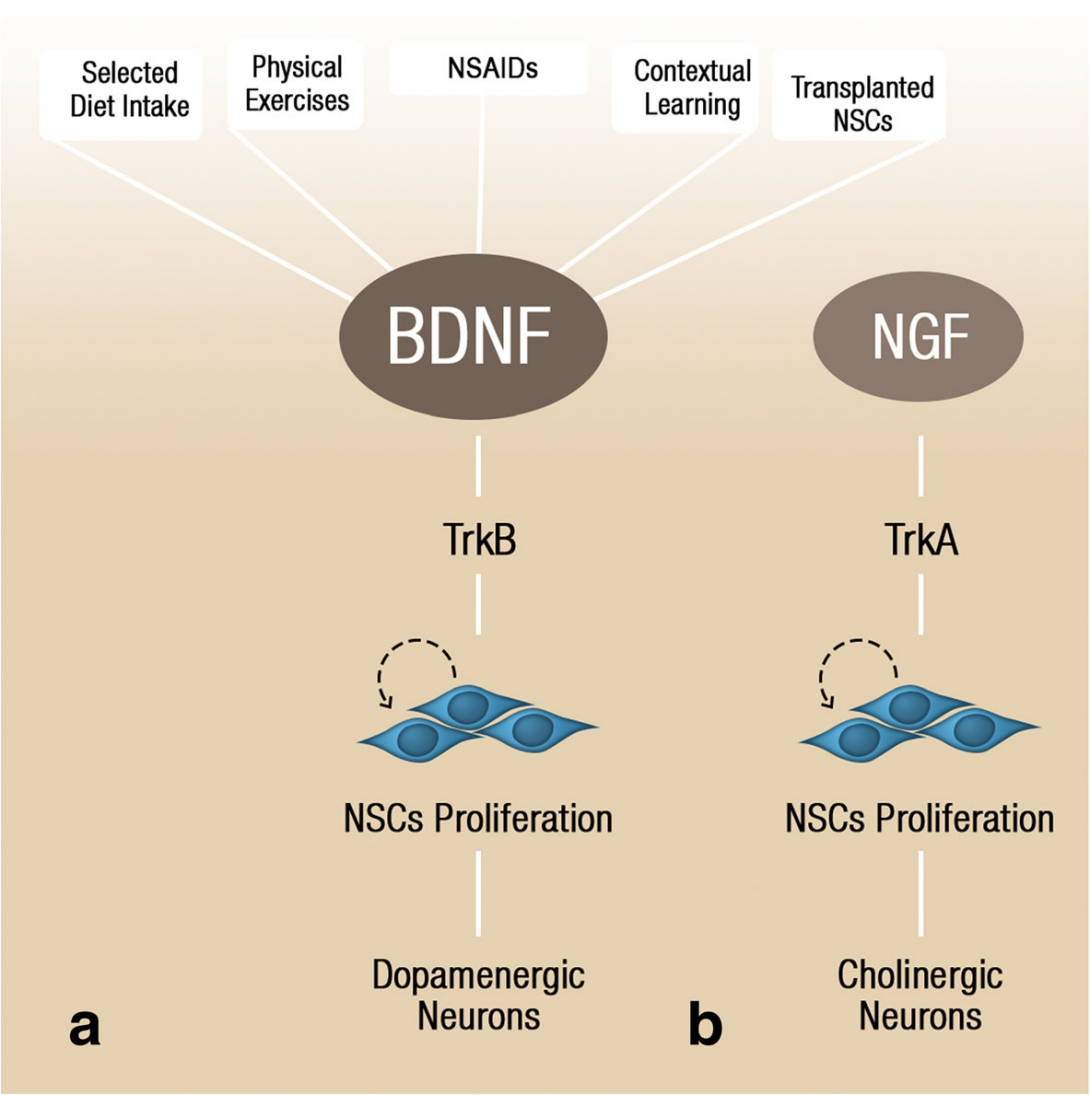
Factors upregulating neurotrophins. BDNF levels and consequently initiate NSC proliferation via activation of the TrkB receptor, which later differentiates into dopaminergic neurons (**a**). NGF, through its downstream receptor TrkA, initiates NSC proliferation that results in cholinergic neurons formation (**b**). The dopaminergic and cholinergic neuronal differentiation occurs primarily during developmental neurogenesis, however, environmental factors, stem cell transplantation, and anti-inflammatory drugs could potentially induce these processes in adult neurogenesis

Endogenous neurotrophic growth factors, which include nerve growth factor (NGF), BDNF, glia-derived nerve factor (GDNF) and insulin-like growth factor 1 (IGF-1), have integral roles in stimulating NSC proliferation, differentiation and central nervous system development [25–27]. At the cellular level, many neurotrophic factors stimulate activation of tropomyosin-related kinase (Trk) receptors, including type A and B, which in turn activates intracellular signaling cascades that regulate NSC self-renewal and fate specification [28]. BDNF-TrkB signaling **(**Fig. 2a**)** was revealed to be essential in the upregulation of hippocampal neurogenesis **(**Fig. 1c**)** and the survival of newly born neurons during adult neurogenesis [29]. Given the essential role of neurotrophic factors in neuronal plasticity and function, a significant number of psychiatric and neurodegenerative disorders are associated with altered neurotrophic factors levels and expression of their cognate receptors [25, 30, 31]. For instance, a reduction in NGF levels and/or deficiency in NGF-TrkA signaling **(**Fig. 2b**)** in cholinergic neurons of forebrain was reported in AD patients and aged rats [32–35], whereas an alteration in BDNF levels was reported in dopaminergic neurons of the substantia nigra in PD patients [36]. Several studies reported that impaired neurogenesis is an early clinical event in both AD and PD [37–39], therefore the lowered levels of neurotrophic factors may account for the observed deficits in neurogenesis. Whilst neurotrophic factors maintain the survival and function of cholinergic and dopaminergic neurons, there are emerging roles for neurotrophic factors, such as BDNF and NGF, in maintaining neurogenesis in the adult brain [40–43]. Therefore, their enhancement in the contexts of neurodegenerative conditions, such as AD and PD, may yield therapeutic benefits.

In order to use neurotrophic factors in therapeutic applications related to aging or neurodegeneration, they have to be delivered *in-vivo* to specific brain regions. In this context, the blood-brain barrier (BBB) represents a considerable challenge for the therapeutic use of neurotrophic factors and their delivery to the brain as neurotrophic molecules are relatively large and polar in nature. In attempts to overcome BBB impermeability, purified neurotrophic factors were directly infused into the brain through intracerebroventricular (ICV) injection. A study, in adult rats, showed that ICV infusion of BDNF induced the generation of new neurons as well as enhancing their survival in the RMS and the OB [40]. Similarly, continuous infusion of NGF through ICV promoted adult hippocampal neurogenesis and new neuron survival in young adult rats [41]. Interestingly, ICV infusion of NGF or BDNF improved cognitive functions in rats as a result of augmented neurogenesis in the hippocampus [42, 44]. Another study in adult rats showed that peripheral infusion of IGF-1 increased progenitor cell proliferation and selectively induced hippocampal neurogenesis [27]. Additionally, in a monkey model of PD, GDNF infusion in the lateral ventricles or the striatum significantly improved motor function, which was associated with upregulation and regeneration of nigral dopaminergic neurons and their innervation to the striatum [45]. Furthermore, a clinical trial in which AD patients received an ICV infusion of NGF reported an improvement in cognitive function [46]. Although NGF infusion in the brain showed slight cognitive improvements in AD patients, long-term administration of NGF through ICV was associated with serious side effects, such as hyper-innervation of cerebral blood vessels [47], hypophagia [48] and neuropathic pain [46]. Taken altogether, these studies indicate that side effects that result from invasive delivery of neurotrophic factors may limit their clinical benefits. In addition, the delivery of clinically beneficial levels of neurotrophic factors has proven challenging.

Further studies have sought to develop approaches that allow the delivery of sufficient levels of neurotrophic factors to the brain. One approach is to engraft cells expressing neurotrophic factors into affected brain regions. A phase I clinical trial of injecting NGF-expressing fibroblasts into the nucleus basalis of AD patients showed sustained NGF expression up to one year following injection and this was accompanied with cognitive improvement [49]. The mechanisms underlying improved cognition were undetermined but an autopsy of a single individual, 5 weeks following implantation, indicated robust growth responses of cholinergic neurons [49]. This study did not evaluate neurogenesis nor did it specifically target the engraftment of NGF-expressing cells to adult neurogenic regions [49]. However, it demonstrated a more sustained expression of neurotrophic factors in comparison to infusion and this was not associated with any long-term adverse effects [49], which indicated that similar strategies could be utilized to specifically augment adult neurogenesis in future trials. One disadvantage of the approach is that it still requires a number of invasive injections directly into the brain that poses a significant risk of neural injury and subcortical hemorrhage [49].

Viral vectors represent an alternative approach for neurotrophic delivery to affected brain regions and to overcome the BBB for neuronal expression of neurotrophic factors. This approach is comparatively less invasive as it requires a single injection of viral particles compared to multiple injections of fibroblasts expressing neurotrophic factors [49, 50]. Adenoviral-mediated delivery of BDNF in normal adult rat brains showed an increase in progenitor cell proliferation in the RMS [51], the OB and the striatum [52], indicative of an increase in the adult neurogenesis. Interestingly, in adult rat brains lesioned with quinolonic acid, adenoviral delivery of BDNF restored progenitor cells proliferation and promoted neuronal differentiation to levels comparable to normal un-lesioned normal brains [51]. In a clinical trial, PD patients who received an intrastriatal infusion of an adenoviral vector expressing aromatic l-amino acid decarboxylase, which enhance dopamine synthesis, demonstrated motor improvements. However, a number of patients experienced intracranial hemorrhage representing a setback in the therapeutic application of viral vectors [53]. Future advances in injection techniques could improve the applicability and reliability of this treatment approach.

Short peptide mimetics are considered a promising therapeutic approach as they target neurotrophic receptors and initiate similar signalling outcomes compare to their full-length counterparts [54]. Moreover, peptide mimetics are highly specific and may potentiate increased receptor activation as they target either TrKA or TrkB receptor with improved bioavailability and lowered proteolysis [54]. The efficacy of these peptide mimetics has been investigated in different animal models. BDNF small peptide mimetic, Peptide B-5, induced the expression of neuronal markers, as well as BDNF and its receptor TrkB, in primary hippocampal neuronal cultures indicating an enhanced neurotrophic effect [55]. Similarly, small peptide mimetics of CNTF, which has established neurotrophic effects [56], have been developed and tested for potential therapeutic benefits [57]. The subcutaneous implant of small peptide mimetics of CNTF, peptide 6, promoted adult neurogenesis in the dentate gyrus, improved neuronal plasticity and spatial memory in mice [57]. The addition of adamantylated glycine groups to CNTF small peptides, which resulted in pentamer named Peptide 021, was also found to significantly increase BBB permeability [58]. The peripheral administration of Peptide 021 induced neurogenesis and neuronal maturation in the hippocampus of adult mice [58]. Further, the enhancement in neurogenesis following Peptide 021 administration was associated with improvements in learning and memory [58]. Another study showed similar outcomes in aged rats with oral administration of Peptide 021 rescuing the age-related reduction in new neurons in the hippocampus [59]. Additionally, Peptide 021 has been shown to increase BDNF and TrkB receptor expression in both the hippocampus and the cortex [59] and, as a consequence, enhance BDNF-dependent adult neurogenesis [40]. Studies in animal models have highlighted the therapeutic potential of neurotrophic mimetics to enhance adult neurogenesis as per their bioavailability, specificity, and minimal side effects. However, additional clinical testing of neurotrophic peptide mimetics is required to reveal their therapeutic benefits.

#### Transcriptional regulation of adult neurogenesis

At the transcriptional level, transcription factors (TFs) regulate the expression of regulatory proteins that play an essential role in promoting adult neurogenesis. Under normal conditions, TFs orchestrate both the proliferation and the differentiation of NSCs into either neuronal or glial lineages [8]. They control the self-renewal of type 1 NSCs in SGZ **(**Fig. 1b**)** and type B NSCs in SVZ **(**Fig. 1b**)** which develop into type 2 cells and type C cells, respectively and the sequence and expression profile of TFs essential for NSC fate determination in adult neurogenesis [60, 61]. The best characterized TFs that have defined functions in regulating adult neurogenesis include: SRY-related high-mobility-group box 2 (Sox2), paired box gene 6 (Pax6), T-box brain gene 2 (Tbr2), RE1 silencing transcription factor (REST), achate-schute complex homolog-like 1 (Ascl1), the orphan nuclear hormone receptor tailless (TLX), cyclic AMP response element-binding protein (CREB), and neurogenic differentiation 1 (NeuroD1) [62–69].

**Fig. 3:**
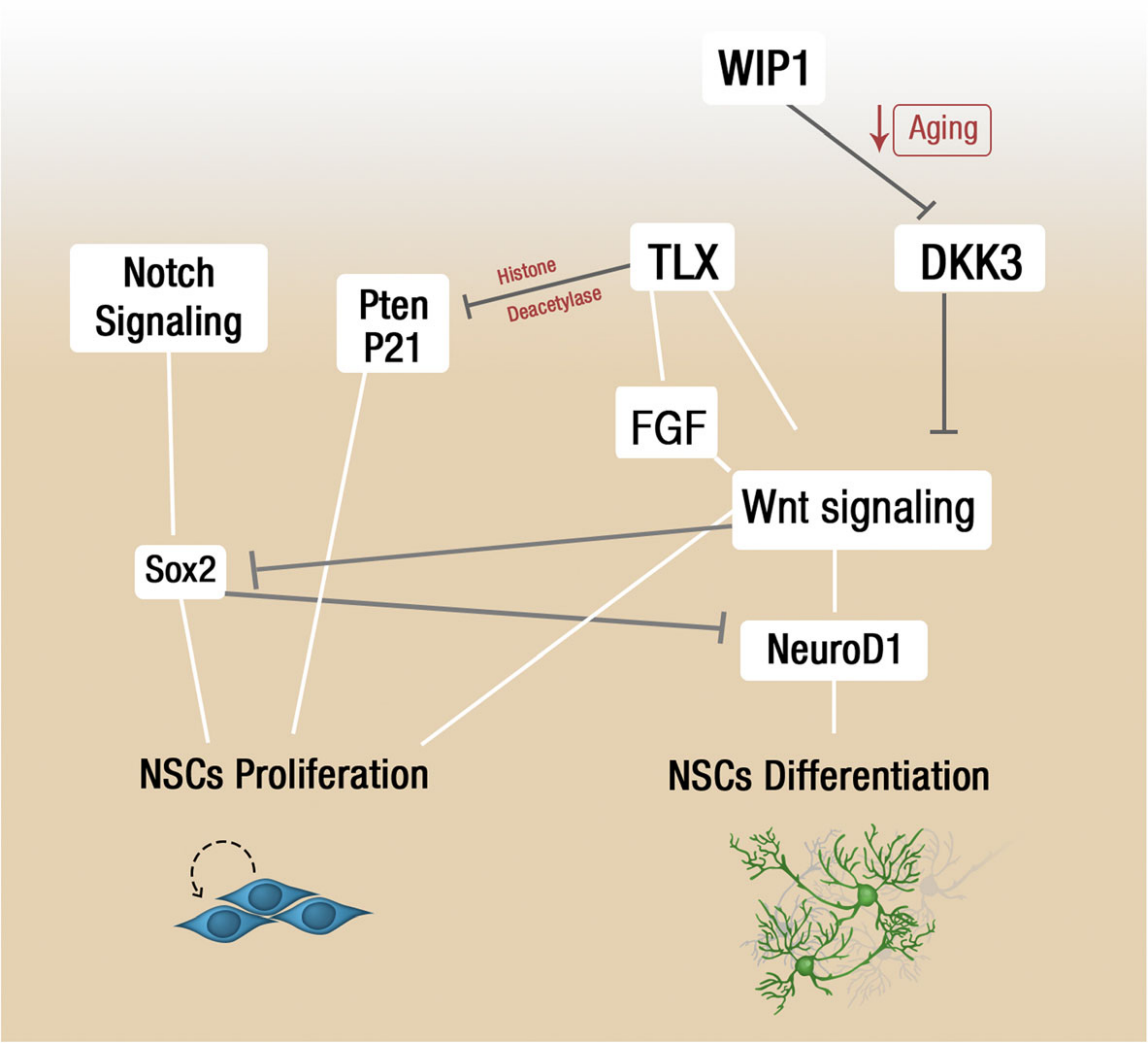
Influence of Notch and WNT signaling on neurogenesis. Wnt signaling enhances NSCs differentiation through the transcriptional upregulation of NeuroD1 and inhibiting Sox2 from restraining NeuroD1 expression. Upon aging WIP1 is downregulated and allow DKK3 to inhibit Wnt signaling that results in reduced NeuroD1 levels and NSC differentiation. In contrast, Sox2, downstream Notch signaling, enhances NSC proliferation. TLX alters the expression of cell cycle regulators as pten, p57, and p21 and induces NSC proliferation

Sox2 is expressed in both radial and horizontal NSCs and play a key role in NSC self-renewal [60, 70]. Its expression is downregulated in postmitotic type 3 neuroblasts as they mature into neurons [71]. The conditional deletion of Sox2 resulted in the depletion of type 1 and type 2 NSCs in the adult SGZ and this was accompanied by a decrease in granule neurons. These findings are consistent with the essential role of Sox2 in maintaining NSC self-renewal and maintaining neurogenic homeostasis [72]. Interestingly, Sox2 interaction with RMST, a long non-coding RNA, was found to be essential in cell fate determination in NSCs [67]. Furthermore, Sox2 was revealed to repress the expression of NeuroD1, a basic transcription factor required for neuronal differentiation and maturation. The removal of Sox2-dependent repression of NeuroD1 by Wnt signaling is required for neurogenesis to proceed [73]. In aged brains, the reduced expression of WIP1 in the aging brain is the result of the increased inhibition of Wnt signaling mediated through Dickkopf 3 (DKK3), a downstream target of WIP1 **(**Fig. 3**)** [74]. This ultimately contributes to the decline of neurogenesis in the aged brain, which raises the interesting prospect that DKK3 could be targeted therapeutically to enhance neurogenesis [74]. Canonical Notch signaling, mediated through the recombination signal binding protein for immunoglobulin kappa J (RBPJκ) pathway, is required to promote and maintain Sox2 expression in NSCs [75]. NSCs are reduced in RBPJκ-deficient animals and rescued by overexpression of Sox2, implicating Notch/RBPJκ-regulated Sox2 as integral for NSC self-renewal [75]. Notch/RBPJκ signaling also promotes the expression of the bHLH gene, Hes5, which (together with Sox2) marks quiescent and actively dividing early progenitor populations in the hippocampus [70]. Pax6 is another important transcription factor that is expressed in the hippocampus and required to sustain the multipotent state of the early progenitors [69]. Pax6 heterozygous rats, which results in reduced postnatal protein expression in the dentate gyrus, also exhibit a reduction in NSC proliferation and the generation of new neurons [69, 70]. Other notable transcriptional regulators of neurogenesis include forkhead box protein O3 (FoxO3) and REST. It has been reported that the absence of the FoxO3 transcription factor in the SVZ and SGZ leads to a failure of NSCs to return to the quiescent state which subsequently causes depletion of the NSC pool [76]. In addition, REST is expressed in type 1 NSCs and type 2 intermediate progenitors and serve to repress the fate commitment of NSCs and the generation of neurons. Its expression is downregulated in type 3 neuroblasts but is re-expressed when cells differentiate into doublecortin-positive immature neurons and subsequently into mature granule neurons [65]. Genetic depletion of REST in NSCs led to a transient increase in neuronal differentiation, which was associated with depletion of the NSC pool and ultimately decreased neurogenic potential in the SGZ. Thus, REST maintains NSCs in a quiescent state by restraining the neurogenic program [65].

Alongside the previously mentioned TFs, TLX is required for maintaining NSCs self-renewal in the SGZ and the SVZ [66, 77]. The mechanism underlying TLX function in adult neurogenesis is subjected to epigenetic control. TLX interacts with HDAC3 and HDAC5 to recruit these histone deacetylases to the promoters of cell cycle regulatory factor p21 and tumor suppressors such as pten and p53 to repress their expression **(**Fig. 3**)** [78]. This, in turn, promotes NSC proliferation [78–80]. It is unclear whether FoxO3, TLX and REST act in concert or function as distinct pathways in regulating adult neurogenesis. TLX was further found to activate Wnt signaling which in turn promotes NSC self-renewal in the presence of epidermal growth factor and fibroblast growth factor [77]. However, as previously mentioned, in the absence of these growth factors, Wnt signaling may also enhance NSCs differentiation through activating NeuroD1 [73, 77]. It is likely that the specific outcome of Wnt signaling may be stimulus-dependent and rely on the context of the neurogenic niche to maintain neurogenic homeostasis. The early progenitors within the adult SGZ and SVZ retain the capacity to specify multiple cell types and it is clear that TF networks determine fate specification and differentiation of newly born neurons. In particular, the expression of the bHLH transcription factor Ascl1, in combination with other TFs, is required for NSCs fate specification. Fate mapping experiments identified glutamatergic interneurons in the OB, which were specified from SVZ progenitors expressing Ascl1 with Neurog2 and Tbr2. The fate of adult progenitors could be manipulated through altered levels and contextual expression of Ascl1 in the NSCs. Retroviral-mediated ectopic Ascl1 expression in the hippocampus resulted in the generation of oligodendrocytes at the expense of granule neurons [81]. These studies raise the intriguing prospect of manipulating NSCs fate specification for therapeutic treatment of neurodegeneration or neural injury.

TFs are also required for the survival and commitment of new neurons in the adult brain. The bHLH TF, NeuroD1, is required for the differentiation and the survival of the neuronal precursors as conditional deletion of NeuroD1 leads to the depletion of new granule neurons and their failure to integrate in the dentate gyrus [64]. NeuroD1 is also required for the terminal differentiation of GABAergic neurons in the OB [82]. The maturation of granule neurons is also dependent on CREB and the homeobox gene Prox1 [68, 83]. Activation of CREB enhances dendritic length and branching of granule neurons. Similarly, Prox1 deletion leads to an arrest in the differentiation of granule neurons [68]. Taken altogether, TFs co-ordinate a complex sequence of events in NSCs during neurogenesis in order to maintain a balance between self-renewal and differentiation. The manipulation of transcription factors may also have utility as a potential therapeutic in neurodegenerative conditions and in preventing neuronal impairment associated with aging. However, there are outstanding questions regarding the specific approach of manipulating transcriptional programs to induce neurogenesis. Since gene expression is dependent on the epigenetic markers on gene promoters, which mediates either repression or induction of genes transcription, one possibility may be through manipulating the epigenetic markers on the promoters of the TFs genes that regulate neurogenesis in the adult brain. Further detailed discussion on this topic was recently elaborated in a recent review by Li *et al.* [84].

#### Neuro-inflammation and adult neurogenesis

**Fig. 4:**
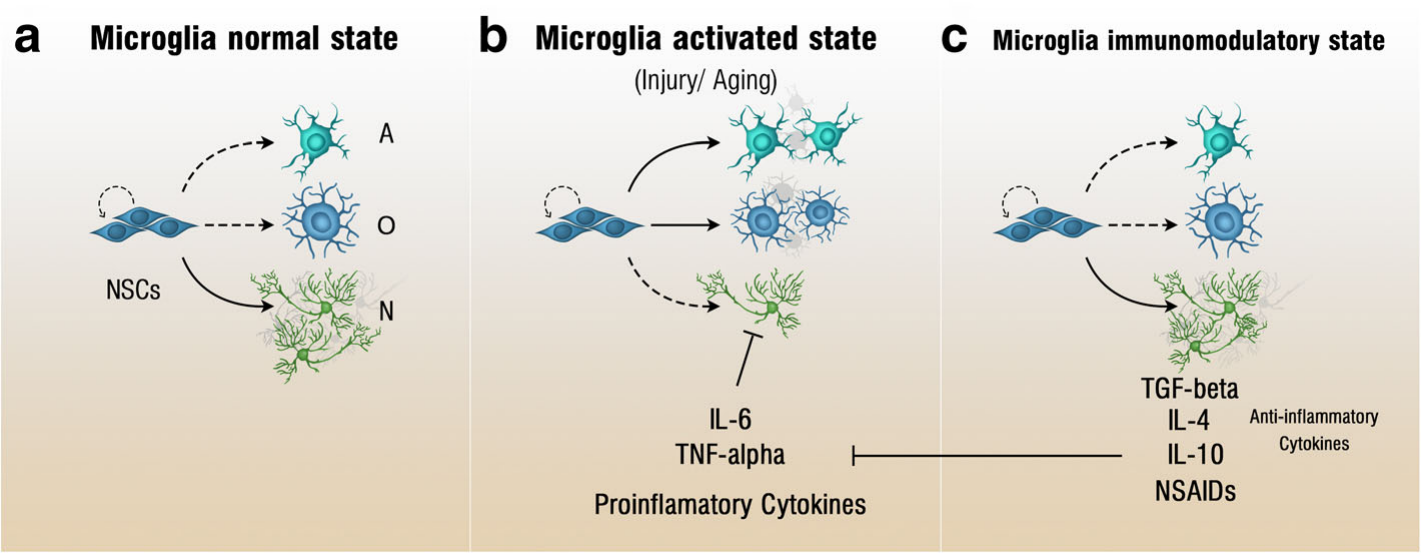
Microglia and neurogenesis modulation. Microglia at the normal state is more likely to undergo neurogenesis rather than gliogenesis (**a**). Upon microglia activation by stress factors such as aging or injury, pro-inflammatory cytokines are released and enhance gliogenesis at the expense of neurogenesis (**b**). In the next stage, microglia reach an immunomodulatory state where anti-inflammatory cytokines are released and a preference for neurogenesis is returned (**c**). ‘A’ - Astrocyte, ‘O’ - Oligodendrocyte, ‘N’ - Neuron

Inflammation was found to play a prominent role in determining the balance of neurogenesis and neurodegeneration [85]. In the brain, microglia are considered the resident macrophages with important immune-regulatory functions in response to brain injury and inflammation [86, 87]. Microglia sense the tissue microenvironment and interact with other cell types in the brain, such as astrocytes and neurons for immune surveillance, as well as maintaining NSCs homeostasis [86, 88, 89]. Microglia **(**Fig. 4a**)** can be classified according to their activation profiles into three distinct phenotypes including resting, activated and alternatively-activated microglia [9, 90]. Microglial activation is known to impinge on the rate of adult neurogenesis [88]. Their impact, however, can vary widely as in the anti-inflammatory state (alternative-activated phenotype) neurogenesis is sustained and supported, while in the pro-inflammatory state (activated phenotype), neurogenesis is diminished [91, 92]. Normally, microglia in the healthy brain is found to be in a resting inactive state [9]. Resting microglia constantly monitor their surrounding microenvironment in order to detect and clear apoptotic cells [93]. The remarkable phagocytic properties of the resting microglia support hippocampal neurogenesis by removing apoptotic neurons and assisting in neuronal integration into the hippocampal circuitry [93]. Walton *et al.* provided further evidence of the regulatory role of the resting microglia in hippocampal neurogenesis by demonstrating that resting microglia release factors that control neuronal differentiation [94]. Using *in vitro* cultured NSCs from the SVZ of 8-day-old mice, they revealed a remarkable depletion in immature neurons over time in the culture, which was related to the decrease in microglia numbers as opposed to NSCs [94]. These studies indicate the important role of microglia in maintaining basal neurogenesis.

In response to brain injury and infection, neuro-inflammation triggers microglia **(**Fig. 4b**)** to attain an activated state, leading to the release of pro-inflammatory cytokines such as tumor necrosis factors α (TNFα), interleukin-6 (IL-6) and IL-1β. These pro-inflammatory cytokines negatively regulate hippocampal neurogenesis by reducing NSC proliferation and neuronal differentiation, in the favor of astrocytes and oligodendrocytes, resulting in a shift towards gliogenesis at the expense of neurogenesis [91, 92, 95–98]. Ekdahl *et al.* reported that brain inflammation, induced by intracortical bacterial lipopolysaccharide (LPS) infusion in rats, increased activated microglia and reduced neurogenesis [99]. Similarly, Monje *et al.* reported a reduction in neurogenesis and a dramatic increase in microglia activation after intraperitoneal injection of LPS in rats with no effect on NSC proliferation [100]. This detrimental effect of neuro-inflammation is mediated through the release of the pro-inflammatory cytokines from activated microglia [9]. For instance, in an *in vitro* study, the pro-inflammatory cytokine IL-6 was suggested to be a key factor released by activated microglia, which in turn reduced neuronal differentiation and induced cell death in existing neurons [100]. Furthermore, it was demonstrated that the overexpression of IL-6 in mice brains reduced proliferation, survival and neuronal differentiation [95]. Likewise, TNFα manifested similar anti-neurogenic effects. Seguin and colleagues reported that systemic administration of TNFα reduced NSC proliferation in the dentate gyrus [101]. Additionally, *in vitro* findings indicated that the addition of TNFα to cultured hippocampal cells resulted in increased cell death [102] as well as an increase in the astrocytic differentiation at the expense of the neuronal differentiation [103]. Furthermore, the addition of IL-1β to cultured hippocampal NSCs reduced cell proliferation and neurosphere formation [104]. Interestingly, the impact of pro-inflammatory cytokines on neurogenesis is not limited to proliferation, cell death, and neuronal differentiation, but may also impact the integration of newly generated neurons in the adult brain [105]. For example, Jakubs *et al.* showed an increase in synaptic plasticity and elevated inhibitory synaptic inputs in newly born neurons matured under a chronic inflammatory environment [105]. Whether this translates to altered brain function remains unclear. Thus, while neuro-inflammation may repress hippocampal neurogenesis, the specific role of microglia in the adult brain is highly dependent on their specific activation profile.

Microglia in the alternatively-activated state **(**Fig. 4c**)** release anti-inflammatory cytokines such as IL-4 and IL-10 as well as transforming growth factor-β (TGF- β), IGF-1 and BDNF, which in turn enhance neurogenesis [91, 98, 106]. Several studies reported that these anti-inflammatory cytokines mediate neuronal differentiation, migration and ultimately neurogenesis [91, 98]. The ectopic expression of the anti-inflammatory cytokine TGF- β via adenoviral vector delivery into the SVZ increased BrdU- and doublecortin-positive cells indicating an enhancement in neurogenesis [107]. Similar findings were seen in neuroprogenitor cell cultures when co-cultured with microglia stimulated with IL-4 [91]. Interestingly, the expression of IGF-1, which has a well-known role in neurogenesis, from microglia was found to increase after being stimulated with IL-4 [108]. Additionally, IL-10-stimulated microglia induced NSC proliferation in culture [109] as well as neuronal differentiation [106]. These findings demonstrate the positive impact of anti-inflammatory cytokines on adult neurogenesis that is mediated through alternatively-activated microglia. Thus, depending on the specific context of activation, microglia may support or conversely restrict neurogenesis [100].

From a therapeutic perspective, anti-inflammatory drugs may partially restore neurogenesis and represent an avenue for adjunct therapy in ameliorating neuronal decline [110]. For instance, Ekdahl and colleagues found that administration of minocycline, an anti-inflammatory drug, restored neurogenesis in the hippocampus and reduced activated microglia [99]. In addition, Kohman *et al.* revealed that minocycline treatment improved spatial learning and neurogenesis in adult mice but not aged ones [92]. In addition, the detrimental effects of peripheral LPS injection on neurogenesis were blocked following the systemic administration of the non-steroidal anti-inflammatory drug (NSAID) indomethacin in rats [100]. This was associated with a reduction in activated microglia and an increase in the number of the newly-born neurons following indomethacin administration [100]. Monje and colleagues have further investigated the effect of indomethacin on microglia activation after cranial irradiation [100]. They showed that indomethacin treatment reduced activated microglia associated with x-ray irradiation induced neural inflammation [100]. An independent study also showed that indomethacin administration one day prior the induction of brain injury via cold light illumination (photothrombosis) elevated the numbers of BrdU- and NeuN-positive cells, which indicates that indomethacin enhanced neurogenesis in the dentate gyrus after brain injury [111]. Clinical treatment with indomethacin and other NSAIDs were able to ameliorate the memory loss in AD patients [112], which might be attributed to the reduction in activated microglia and enhanced neurogenesis. Clinical trials are required to investigate the efficiency of anti-inflammatory drugs treatment and anti-inflammatory cytokines administration, such as IL-4 and IL-10 that are predicted to modulate microglial activation profiles in favor of neurogenesis. The long-term use of such treatments may have significant side effects, which should also be investigated and considered. This will determine the broad utility of anti-inflammatory approaches for the restoration of neurogenesis during brain injury or neurodegeneration.

#### Neurotransmitters in adult neurogenesis

Neurotransmitters such as glutamate, gamma-aminobutyric acid (GABA), acetylcholine (Ach), dopamine and serotonin (5-HT) mediate neuronal communication but are further implicated in neurogenesis in development and in adult brains. We direct the reader to recent reviews dedicated to specific neurotransmitters for detailed summaries of their role in the regulation of neurogenesis [113–116]. Here, we discuss key findings pertinent to our current discussion, that is direct neurotransmitter effects on adult NSCs and pharmacological studies that have attempted to augment adult neurogenesis *in vitro* and *in vivo*.

It is well established that neurotransmitters influence proliferation and differentiation of cells within neurogenic zones. NSCs in the SVZ express glutamate receptors (NMDA, kianate, metabotropic glutamate receptors [mGluRs]) where glutamate signalling can promote cell proliferation [117, 118]. Excitatory stimulation of NMDA receptors on adult hippocampal NSCs result in elevated intracellular calcium and activation of the proneural gene, NeuroD1, highlighting direct effects of glutamate on adult neuroprogenitor cells [115]. Studies that have utilized genetic deletion of NMDA receptor subunits or pharmacological blockage of mGluR1 have supported a positive role for glutamate signalling in maintaining the proliferation rates of NPCs and survival of newly generated neurons [117, 119]. These studies were performed on *in vitro* NSC cultures or utilized single-cell knockout approaches to indicate cell-type specific contribution of glutamate receptor activation in NSCs. Similarly, NSCs in the SGZ and SVZ express, specifically, the GABA_A_ receptor subtype [120–122]. The paracrine activation of GABA_A_ receptors on NSC populations by their progeny has been shown to have a non-synaptic inhibitory effect on proliferation and likely function as a negative feedback mechanism to modulated proliferation in adult and post-natal brains [120, 121]. In support of this, the *in vivo* administration of GABA_A_ receptor agonists (phenobarbital) reduced NSC proliferation and increased differentiation leading to enhanced numbers of newly generated neurons [123]. Furthermore, genetic deletion of specific GABA_A_ receptor subunits have indicated spatiotemporal-specific functions in regulating proliferation, migration and maturation processes that constitute adult hippocampal neurogenesis [122, 124].

Dopamine, serotonin (5-HT) and acetylcholine (Ach) are neurotransmitters with important neurobehavioural functions including the regulation of mood, behavior, learning and memory. It is also increasingly appreciated that their role in regulating adult neurogenesis may be a contributing factor in pathologies associated with the decline of these neurotransmitters in aged and diseased brains. The ablation of dopaminergic neurons with 6-hydroxydopamine resulted in decreased proliferation in the SVZ and SGZ suggesting that decreased neurogenesis may contribute to the progression of PD [125]. In support, post-mortem studies of brains from PD patients have reported reduced proliferation in the SVZ [125]. The infusion of the dopamine precursor, levodopa, reversed ablation-induced proliferation deficits in the SVZ in animal models of PD [125, 126]. However, direct agonist stimulation of dopaminergic receptors present on NSCs in the SVZ, to promote adult neurogenesis, may represent the most clinically promising approach for PD patients when taking into consideration the substantial loss of dopaminergic neurons at the time of disease diagnosis [113, 127].

Similarly serotonergic neurons project from the raphe nucleus in the dentate gyrus while loss of 5-HT by neurotoxic lesions or synthesis inhibition results in reduced hippocampal proliferation that is rescued by implantation of fetal raphe neurons expressing 5-HT [128–130]. Pharmacological studies that have manipulated 5-HT levels have generally supported a positive effect on adult neurogenesis. For example, systemic administration of 5-HT receptor agonists or fluoxetine, a selective serotonin reuptake inhibitor, increased cell proliferation and newly born neurons in the SVZ and dentate gyrus [130, 131]. Whether these effects are mediated through direct effects of 5-HT receptors on NSCs is unclear. There are also a number of 5-HT receptors and their cell-specific expression patterns and functions in the neurogenic zones are not fully resolved [118]. Altered 5-HT levels are associated with depression and anxiety. However further studies are required to determine the extent to which a decline in neurogenesis contributes to mood disorders. Nevertheless Santarelli et al. demonstrated that anti-depressive effects of fluoxetine or imipramine were lost following x-ray ablation of cell proliferation in the SGZ indicating an important contribution of adult neurogenesis in the mood-modulating effects of 5-HT [132].

Acetylcholine (Ach) is critically required for learning and memory functions in the hippocampus which is also known to require adult neurogenesis [125]. Importantly, altered Ach levels are linked to neurodegeneration, specifically AD where the disease severity in patients correlates with decreases in cholinergic tone [133]. The best evidence of the positive effects of Ach on adult neurogenesis have come from studies that have performed cholinergic forebrain lesions which resulted in decreased proliferation, survival of NSCs, and new neurons in the dentate gyrus as well as deficits in learning and memory [134, 135]. In addition, increases in extracellular Ach following intraperitoneal administration of donepezil, a cholinesterase inhibitor, in mice induced the survival of newly born neurons in the dentate gyrus [127]. There is less evidence of direct cholinergic inputs into the SVZ [127]. NSCs express a number of Ach receptor subtypes (muscarinic and β2-nicotinic receptors) and in vitro studies indicated muscarinic receptor signalling as required for embryonic NSC proliferation [136, 137]. However, more detailed studies are required to determine the signalling mechanisms underlying the Ach regulation of adult hippocampal neurogenesis and their functions in learning and memory.

Taken together these studies indicate important neurotransmitter regulation of adult neurogenesis. Studies in animal disease models also suggests the potential for pharmacological manipulation of neurotransmitter levels and/or receptor activation during pathological brain states to enhance adult neurogenesis.

#### Influence of hormones on adult neurogenesis

Hormones are signalling components of the neuroendocrine system with integral functions in regulating human physiology and behavior. Accumulating evidence point to their role as intrinsic factors that modulate adult neurogenesis and neuronal plasticity [10]. In this section, we discuss hormonal influence on adult neurogenesis with a focus on sex hormones, glucocorticoids and specific metabolic hormones.

Androgens (including testosterone), estrogen and progesterone are the predominant gonadal sex hormones expressed by the testes or ovaries. There is a relationship between fluctuations in the levels of sex hormones and proliferation in the hippocampus. For example, peak levels of ovarian hormones during proestrus is correlated with increased levels of proliferation in the SGZ [138, 139]. The reductions in testicular hormones during stages of reproductive inactivity in some animals are also associated with reduced production of new neurons [140]. In addition, sex hormone levels, which are highly expressed early in life and decline with age, coincide with reduced proliferation and survival of newly generated neurons [141]. This raises the question of whether the age-related decline in neurogenesis may be related to natural reductions in hormone levels. In rats, the surgical removal of ovaries, the main source of estrogens, resulted in reduced proliferation in the hippocampus at 6-7 days following ovariectomy [141]. The acute exposure of exogenous estradiol has also been reported to rescue the reduction in hippocampal proliferation following ovariectomy although these effects are highly dependent on dose, duration of hormone exposure and time of analysis post-surgery [141]. The influence of estrogen on hippocampal cell proliferation is mediated through estrogen receptors (ER) as estradiol-induced cell proliferation in the hippocampus can be inhibited by ER antagonist [142], whereas ER agonist enhances cell proliferation [143]. Progenitor cells in the dentate gyrus express ERs so estrogen effects on adult neurogenesis may be mediated by direct hormone effects on NSCs [143]. Interestingly, hippocampal cell proliferation is restored at 4 weeks following ovary removal in rats [142]. Hojo et al. reported synthesis of estradiol by hippocampal neurons [144]. This extra-gonadal source of estrogen may explain the restoration of hippocampal proliferation at extended durations following ovariectomy although the precise underlying mechanisms remain undetermined [141]. In contrast to estrogen, the depletion of androgens by castration in adult male rats did not impact proliferation in the hippocampus but reduced the survival of new neurons [145]. Pharmacological inhibition or genetic inactivation of androgen-receptors also support testosterone effects on neuron survival as mediated through androgen receptor-dependent signaling [146]. However, androgen-receptors are absent from the dentate gyrus which suggests that testosterone effects on new neuron survival may involve indirect mechanisms [140].

The acute and chronic exposure to psychosocial stress in various animals may also repress neurogenesis in the dentate gyrus [144]. These negative effects are thought to be mediated through stress gluococorticoids [147]. The chronic administration of corticosterone, for example, reduced cell proliferation and the density of immature neurons in the adult hippocampus in both male and female rats [148]. Furthermore, the depletion of glucocorticoids, through removal of the adrenal gland, resulted in an increase in adult hippocampal neurogenesis and removed stress-induced suppression of cell proliferation in the hippocampus [145]. Neural-restricted deletion of the gluococorticoid receptor prevented stress-induced decreases in hippocampal neurogenesis which highlight glucocorticoid receptor activation as mediating the negative influence of stress hormones on neurogenesis [149]. As glucocorticoid receptors are expressed throughout the brain, further studies are required to delineate direct effects of stress hormones on NSCs.

Metabolic hormones, such as leptin and incretin, have reported roles in modulating hippocampal neurogenesis [147]. Chronic administration of leptin enhanced cell proliferation in the hippocampus but did not impact differentiation or survival of new neurons [150]. Similarly, agonist stimulation of incretin hormone receptors enhanced proliferation and generation of new neruons in the dentate gyrus [151]. Furthermore, the 8 week administration of liraglutide, an analog of the incretin hormone glucagon-like peptide-1, prevented the decline in hippocampal neurogenesis in a mouse model of AD [152]. Non-human primates with low body mass index and high GLP-1 levels also tend to have higher hippocampal neurogenesis in comparison to those with high body mass index and low GLP-1 levels [153]. These studies highlight metabolic hormone influences on adult hippocampal neurogenesis. Further studies could determine if manipulation of hormone levels may be used to intervene in disease or age-related decline in neurogenesis.

### Extrinsic factors that regulate adult neurogenesis

#### Physical activity impact on adult neurogenesis

There is a robust association between physical exercise and adult neurogenesis, as physical activity is known to upregulate neurogenesis in the SGZ and the SVZ [154–157]. Early studies that characterized and described this association were carried out by Van Praag *et al.* in mice. They showed that neither swimming, nor maze training improved cell proliferation and neurogenesis in the dentate gyrus, however, a voluntary exercise in a running wheel doubled the number of proliferating cells and net neurogenesis in the dentate gyrus [157]. In contrast, an independent study showed that exposing rats to regular swimming exercise resulted in an increase in the progenitor cell proliferation and maturation in the SVZ [155]. Species specific differences, that is rats instead of mice, and also the frequency or the duration of swimming exercise may account for the discrepancies in findings on the extent of physical activity required to elicit augmented neurogenesis. Interestingly, the increase in the SVZ neurogenesis in rats after regular swimming exercise was associated with an increase in the trophic factor NGF, which may contribute to inducing neurogenesis following physical activity [155]. Other work carried out by Kronenberg *et al.* showed that sustained running in mice resulted in an acute but transient increase in NSC proliferation [154]. Moreover, the number of doublecortin-positive immature neurons increased significantly with continued running, despite a return of NSC proliferation rates to baseline levels in the dentate gyrus [154]. This increase in neurogenesis in the dentate gyrus following exercise was associated with an enhancement in the spatial memory, suggesting that consistent exercise may improve cognitive function due to augmented neurogenesis [158]. In agreement, Shors *et al.* revealed that newly-born neurons contribute towards acquiring tracing memory [159]. Furthermore, physical activity may preserve neuronal plasticity and improve learning as mice demonstrated enhanced performance in water maze tests following wheel running [160]. In support of this, inhibition of neurogenesis by focal irradiation removed exercise-stimulated increases in spatial learning [161]. These studies highlight the links between exercise and hippocampal neurogenesis and how this could be reflected in improved cognitive function.

Physical activity enhances hippocampal neurogenesis and cognitive function through promoting the increase in the cerebral blood flow [162], BBB permeability [163], angiogenesis [164] and the expression of neurotrophic factors [10]. In the following we discuss the link between neurotrophic factors as mediators of the effects of physical activity in inducing neurogenesis. It has been demonstrated that physical activity increased levels of neurotrophic factors, such as NGF [155], IGF-1 [165, 166], vascular endothelial growth factor (VEGF) [167], and BDNF [168]. The augmented release of these neurotrophic factors may underlie the ability of exercise to enhance adult neurogenesis.

For instance, Lafenêtre *et al.* generated mice that were genetically modified to have down-regulated cell proliferation in the hippocampus and short-term memory [169]. After running, these mice showed a reverse of a genetic blockade of cell proliferation and improved short-term memory [169]. An increase in BrdU- and doublecortin-positive cells were seen in the hippocampus mice after running [169]. Interestingly, an upregulation in BDNF receptor, TrKB, was seen in doublecortin-positive cells following running, which may signal the increase in hippocampal neurogenesis [169]. The inhibition of IGF-1 signaling, using a specific antibody targeting the IGF-1 receptor, resulted in the loss of exercise enhanced cognition in rats [170]. Furthermore, it has been reported that running exercise enhanced IGF-1 uptake by specific neurons in the rat brain, which resulted in a spontaneous firing of neurons and elevated the expression of BDNF [171]. In addition to BDNF and IGF-1, VEGF has been shown to have a neurotrophic effect and increased levels have been observed following exercise in rats [172]. Peripheral blockade of VEGF prevented augmented neurogenesis induced by running, whereas VEGF blockade did not alter baseline neurogenesis in non-running mice [173] which indicates VEGF contributions to exercise-mediated adult neurogenesis. In a clinical study, investigation of high to moderate intensity exercise groups revealed an increase in BDNF and IGF-1 levels that were associated with enhanced cognitive function when compared to low-intensity exercise groups [174]. These studies highlight the increased production of growth factors as underlying the beneficial neurophysiological effects of physical activity. As a result, regular physical exercise could be useful as a non-invasive approach of inducing the endogenous expression of neurotrophic factors, which ultimately induce neurogenesis.

#### Dietary role in adult neurogenesis

Dietary intake represents a modifiable behavior and an extrinsic factor that can alter cognitive function and adult neurogenesis [11]. Dietary behavior including dietary restriction or intake of certain diet textures or content have been reported to influence adult hippocampal neurogenesis [175]. According to Lee and colleagues, dietary restriction for 4 weeks in rodents resulted in increased BrdU- and NeuN-positive cells in the dentate gyrus, which indicates an increase in proliferation and neuronal differentiation, respectively [176, 177]. Lee *et al.* have further revealed that the impact of the dietary restriction on hippocampal neurogenesis was mediated by BDNF [7]. In this study, they compared BDNF +/+ control mice and BDNF -/+ heterozygous mice maintained on *ab libitum* diet (normal diet according to animal needs) or a regimen of dietary restriction for 3 months [7]. After 4 weeks of BrdU injection, BrdU-positive cells were decreased in BDNF-/+ mice maintained on *ab libitum* diet, however, dietary restriction significantly increased BrdU-positive cells in the dentate gyrus of BDNF +/+ mice and to a lesser extent in BDNF -/+ mice [7]. This indicates a positive effect of dietary restriction on NSC proliferation that involves BDNF levels [7]. In addition, IGF-1 is a neurotrophic factor that has also been suggested to be elevated in rodents maintained on a restricted diet regimen [178]. A number of studies have also reported that the effects of dietary restriction extend to behavioral and cognitive functions [179, 180]. Therefore, combined exercise and dietary normalization had positive additive effects in enhancing BDNF and NGF expression and improving decreased cognitive function in high fat-induced obese adult rats [181]. Pitsikas *et al.* reported that dietary restriction has significantly improved learning and memory in aged rats [180]. In humans, a study on a cohort of 49 healthy elderly subjects reported that the subjects maintained on a regimen of dietary restriction, in comparison to subjects maintained on *ab libitum* diet, exhibited enhanced verbal memory, albeit without increased serum levels of BDNF [182]. This does not necessarily exclude BDNF from mediating the improved memory of elderly subjects maintained on dietary restriction as peripheral BDNF levels were reported and neural BDNF levels remained unknown [182]. In addition to caloric restriction, some reports suggest that diet texture can alter neurogenesis in the hippocampus [183]. This was first reported by Aoki and colleagues who demonstrated that rats on soft-diet feeding led to reduced proliferation in the hippocampus [184]. However, a reduction in proliferation may not necessarily translate to reduced neurogenesis as Patten *et al.* similarly reported a decrease in hippocampal proliferation in rats fed a liquid diet but did not observe significant changes in the differentiation and survival of new neurons compared to groups fed a solid diet [185]. Since cell proliferation was decreased, Patten and colleagues postulated that compensatory mechanisms were involved in maintaining hippocampal neurogenesis [185]. However, other studies have reported inhibitory effects on neurogenesis in mice [183, 186]. In this context, mice fed a soft-textured diet exhibited reduced proliferation and neurogenesis in the hippocampus, when compared to hard-diet feeding of equivalent calories [183, 186]. Furthermore, mice fed on hard-diet recovered the impairment in the olfactory function resulted from soft-diet feeding, which was related to the enhancement in neurogenesis [186]. Interestingly, the inhibition in the hippocampal neurogenesis after soft-diet feeding was linked to a down-regulation in BDNF expression following a reduction in the mastication activity [187]. Thus, the improvement in the hippocampal neurogenesis in rodents fed on hard-diet may be attributed to the physical act of mastication [175]. These studies indicate the potential of dietary restriction and diet texture in enhancing the hippocampal neurogenesis through upregulating neurotrophic factors.

Nutritional content may also represent a dietary factor that can influence adult neurogenesis. Diets high in saturated fats and simple sugars can dramatically impair neurogenesis, learning, and memory in rodents and lower BDNF levels expression [188]. In contrast, diets rich in poly-unsaturated fatty acids (PUFA) and polyphenols induce neuronal plasticity in the adult hippocampus [11]. Supplementation of PUFA in rodents increased proliferation, NSC differentiaton into neurons in the dentate gyrus and increased hippocampal volume, which indicates an increase in the hippocampal neurogenesis [189–191]. The enhancement in hippocampal neurogenesis after PUFA supplementation was also associated with an increase in cognitive function and learning [190]. Interestingly, BDNF levels were found to increase after PUFA supplementation in mice, which may mediate PUFA-induced neurogenesis in the hippocampus [191]. In addition to PUFA, polyphenols (flavonoids and other subtypes) are additional dietary compounds that may modulate adult hippocampal neurogenesis [11]. The flavonoid resveratrol, enriched in the skin of red grapes, amongst other fruits and plants, was shown to enhance hippocampal neurogenesis through up-regulating CREB levels to subsequently promote BDNF synthesis in the hippocampus [192]. Furthermore, Harada *et al.* showed that oral administration of resveratrol in mice elevated IGF-1 levels in the hippocampus through stimulation of gastrointestinal tract sensory neurons, thereby improving neurogenesis and cognitive function [193]. In addition, blueberries are rich in the flavonoid anthocyanin and other polyphenol subsets, which were able to reverse the neuronal and behavioral impairment related to aging in rats [194]. Blueberry supplementation in rats resulted in an enhancement in neuronal plasticity and improved cognitive function that was associated with increased IGF-1 and BDNF levels [195, 196]. Similarly, the flavonoids quercetin and kaempferol were found to elevate hippocampal BDNF expression in mice to promote neuronal plasticity [197]. Curcumin, found in turmeric [198], is another polyphenolic compound that was reported to improve learning and memory in aged rats [199, 200]. Dong *et al.* reported that curcumin supplementation for 12 weeks in aged rats enhanced hippocampal neurogenesis [200]. According to an epidemiological study, elderly subjects with diets comprising a large amount of turmeric showed an improvement in cognitive function compared to elderly subjects with diets low in turmeric [201]. Thus, manipulating dietary content and intake may be one approach to enhance adult neurogenesis. In addition, these dietary studies also highlight naturally occurring compounds that could potentially be exploited therapeutically to enhance cognitive function in clinical settings.

As previously explained; BDNF levels, and other neurotrophic factors, were altered in response to different extrinsic factors including diet and physical exercises **(Fig.** **2****A)**, which in return mediate hippocampal neuronal plasticity. Hence, defined nutritional intake and regular physical exercises are recommended lifestyle approaches to enhance neurotrophic factors expression and maintain NSCs homeostasis in the hippocampus. These lifestyle interventions may serve to prevent, or significantly improve the status of neurogenesis in aging and neurodegeneration.

#### Stem cell therapy and adult neurogenesis

The discovery of NSCs with persistent proliferative capacity in adulthood have challenged the dogma that the adult brain is incapable of regeneration. Although some studies have explored the regenerative potential of endogenous NSCs following stroke injury [202–204], it is clear that endogenous neurogenesis is insufficient in some disease contexts, for example, in chronic conditions. Thus, augmenting neurogenesis through stem cell transplantation represents an area of intense investigation as an approach to replace lost neurons caused by neural degeneration or injury. Qu *et al.* reported that the injection of human NSCs in the lateral ventricles of aged rats improved cognitive function in aged animals as a result of the ability of the transplanted NSCs to differentiate into neuronal lineages in the hippocampus including neurons and astrocytes [205]. Similarly, Lee and colleagues showed that intravenous injection of human NSCs into striatum-lesioned rats reduced atrophy and this was attributed to the migration of transplanted NSCs to striatal lesions and subsequent differentiation into neurons and astrocytes [206]. Another study utilizing the nucleus basalis of Meynert lesion mouse model of AD demonstrated that the transplantation of mouse NSCs-derived neurospheres into the frontal region of the cortex reversed cognitive deficits [207], as transplanted neurospheres were able to produce cholinergic and serotonin-positive neurons around the grafts and integrated into the cerebral cortex [207]. Furthermore, in parkinsonian model, the stereotaxic injection of human NSCs into the substantia nigra resulted in improvements in behavior and movement, which was linked to the capacity of the transplanted NSCs to differentiate into tyrosine hydroxylase- and dopamine transporter-positive cells in the substantia nigra [208]. More recently, a study in a PD mouse model showed engraftment of induced NSCs (converted from mouse embryonic fibroblasts) resulted in differentiation into dopaminergic neurons and migration to the substantia nigra [209].

An additional consideration following transplantation is promoting the migration and integration of transplanted cells to the infarct regions of the brain after injury [210]. In this context, Imitola *et al.* showed that in response to ischemia in mouse brain, astrocytes and endothelial cells up-regulate the expression of the inflammatory chemoattractant stromal cell-derived factor 1α (SDF-1α) [210]. SDF-1α stimulates the cognate receptor, CXC chemokine receptor 4 (CXCR4), expressed on the NSCs to promote their proliferation and migration towards infarct regions [210]. More recent studies also provided evidence that SDF-1α/CXCR4 promoted mobilization and homing of exogenously transplanted NSCs to sites of injury in mouse brains [211, 212]. Thus, the optimization of SDF-1α expression in the brain of the host microenvironment may potentially improve the migration of endogenous NSCs or even transplanted cells and re-direct them towards the sites of the brain injury. These studies demonstrate the potential of transplanted NSCs in augmenting neurogenesis to produce neurons that integrate into different brain regions during aging and neurodegeneration. However, the specific impact of stem cell transplantation on endogenous NSC populations is less well understood but may contribute significantly to overall cognitive benefits.

Some studies suggest that transplanted stem cells may impact endogenous neurogenic mechanisms. Blurton-Jones *et al.* found that the ability of transplanted NSCs to differentiate into neuronal lineages and integrate into the hippocampus, as well as the improvement in cognitive function in mice, was due to the elevated expression of BDNF mediated through NSCs transplantation [213]. Interestingly, other studies in rodents reported that mesenchymal stem cells (MSCs) transplantation induced neurogenesis in the hippocampus [214] and improved cognitive functions [215, 216]. *In vitro* studies revealed that MSCs express BDNF, NGF, and IGF-1, which could potentially mediate neurogenesis in the hippocampus [217, 218]. Tfilin *et al.* reported that *in vivo* injection of MSCs in the brain ventricles of rats resulted in an increase in the hippocampal neurogenesis, which was mediated through an elevation of BDNF expression in the hippocampus [218]. Additionally, Yang and colleagues have investigated the beneficial effects of MSCs transplantation towards neurogenesis and cognitive function [215] and they reported that alternatively-activated microglia were significantly increased after MSCs transplantation in mice [215]. This state of microglial activation resulted in elevated levels of the anti-inflammatory cytokine IL-4 and decreased levels of the pro-inflammatory cytokines, including IL-1β and TNF-α [215]. This alteration in the neuro-inflammation following MSCs transplantation may promote the potentiation of neurogenesis following MSC transplantation. Furthermore, Oh *et al.* showed that intravenous injection of MSCs in mice induced neurogenesis that was due to the upregulation of Wnt/β-catenin signaling [214]. Taken altogether, these studies highlight the potential of translating stem cells for cognitive benfefits in aging and neurodegeneration. Currently, there are seven ongoing clinical trials investigating the safety and the efficacy of MSCs therapy in humans [219]. The majority of trials have adopted intravenous delivery for stem cell administration as this is much less invasive than intracranial injections [219]. The completion of these clinical studies will help address the potential of stem cell therapy in humans.

## Conclusion

Despite extensive studies in animal disease models, the therapeutic benefits of adult neurogenesis has yet to be realized in human trials. However, our knowledge of neurogenic mechanisms and the factors that determine adult neurogenesis has expanded greatly in the last few decades. There is a considerable advance in our understanding of the roles of the vasculature and astrocytes within the neurogenic niche and how signalling from the niche is integrated with several extrinsic factors including dietary intake and physical activity. These extrinsic physiological factors likely modulate neurogenesis through a complex network of neurotrophic factors, TFs, inflammatory cytokines, neurotransmitters, and hormones. It is conceivable that a well-defined ‘proneurogenic’ lifestyle could delay the occurrence of neurodegeneration associated with aging or even improve the status of neurodegenerative patients. Interestingly, neurogenesis and neural plasticity were enhanced with neurotrophic small mimetics, however, this requires further investigation and clinical testing in humans. Additionally, advanced approaches in the minimally invasive delivery of neurotrophic factors expressing viral vectors and stem cells may result in further improvements in augmented neurogenesis as a therapy. Lastly, the manipulation of neurotransmitters and hormonal activity in the brain may also have utility in potentiating adult neurogenesis for therapeutic benefits. Future studies will reveal the clinical applicability of such approaches as a therapy in aging and neurodegenerative conditions.

## Abbreviations

5-HT: 5-hydroxytryptamine
Ach: Acetylcholine
AD: Alzheimer’s disease
Ascl1: Achate-schute complex homolog-like 1
BBB: Blood-brain barrier
BDNF: Brain-derived neurotrophic factor
CNTF: Ciliary neurotrophic factor
CREB: Cyclic AMP response element-binding protein
CXCR4: CXC chemokine receptor 4
DKK3: Dickkopf 3
ER: Estrogen receptors
GABA: Gamma-aminobutyric acid
GDNF: Glial-derived nerve factor
GLP-1: Glucagon-like peptide-1
ICV: Intracerebroventricular
IL: Interleukin
LPS: Lipopolysaccharide
mGluRs: Metabotropic glutamate receptors
MSCs: Mesenchymal stem cells
NeuroD1: Neurogenic differentiation 1
NGF: Nerve growth factor
NSAIDs: Non-steroidal anti-inflammatory drugs
NSCs: Neural stem cells
OB: Olfactory bulb
Pax6: Paired box gene 6
PD: Parkinson’s diseases
PUFA: Poly-unsaturated fatty acids
RBPJκ: Recombination signal binding protein for immunoglobulin kappa J
REST: RE1 silencing transcription factor
RMS: Rostral migratory stream
SGZ: Subgranular zone
Sox2: SRY-related high-mobility-group box 2
SVZ: Subventricular Zone
Tbr2: T-box brain gene 2
TFs: Transcription factors
TGF- β: Transforming growth factor-β
TLX: Orphan nuclear hormone receptor tailless
TNFs: Tumor necrosis factors
Trk: Tropomyosin-related kinase
VEGF: Vascular endothelial growth factor
